# Pyramidal Neurons in Different Cortical Layers Exhibit Distinct Dynamics and Plasticity of Apical Dendritic Spines

**DOI:** 10.3389/fncir.2017.00043

**Published:** 2017-06-19

**Authors:** Michelle Tjia, Xinzhu Yu, Lavpreet S. Jammu, Ju Lu, Yi Zuo

**Affiliations:** Department of Molecular, Cell and Developmental Biology, University of CaliforniaSanta Cruz, CA, United States

**Keywords:** dendritic spines, spine plasticity, *in vivo* imaging, motor-skill learning, sensory deprivation

## Abstract

The mammalian cerebral cortex is typically organized in six layers containing multiple types of neurons, with pyramidal neurons (PNs) being the most abundant. PNs in different cortical layers have distinct morphology, physiology and functional roles in neural circuits. Therefore, their development and synaptic plasticity may also differ. Using *in vivo* transcranial two-photon microscopy, we followed the structural dynamics of dendritic spines on apical dendrites of layer (L) 2/3 and L5 PNs at different developmental stages. We show that the density and dynamics of spines are significantly higher in L2/3 PNs than L5 PNs in both adolescent (1 month old) and adult (4 months old) mice. While spine density of L5 PNs decreases during adolescent development due to a higher rate of spine elimination than formation, there is no net change in the spine density along apical dendrites of L2/3 PNs over this period. In addition, experiences exert differential impact on the dynamics of apical dendritic spines of PNs resided in different cortical layers. While motor skill learning promotes spine turnover on L5 PNs in the motor cortex, it does not change the spine dynamics on L2/3 PNs. In addition, neonatal sensory deprivation decreases the spine density of both L2/3 and L5 PNs, but leads to opposite changes in spine dynamics among these two populations of neurons in adolescence. In summary, our data reveal distinct dynamics and plasticity of apical dendritic spines on PNs in different layers in the living mouse cortex, which may arise from their distinct functional roles in cortical circuits.

## Introduction

The mammalian cerebral cortex plays an essential role in perception, motor control and higher cognitive functions. It consists of distinct areas, which are dedicated to specific functions but share a common laminar structure. Neurons in different cortical layers can be classified into subtypes, the most abundant being the pyramidal neurons (PNs; DeFelipe and Fariñas, 1992). PNs are glutamatergic excitatory neurons (DeFelipe, 2011); they usually have pyramid-shaped somata and communicate with other cortical or sub-cortical regions of the brain via long-distance axonal projections (DeFelipe and Fariñas, 1992; Spruston, 2008).

PNs located in different cortical layers vary considerably in their connectivity, dendritic morphology and functional properties (Feldmeyer, 2012; Harris and Shepherd, 2015). First, their axons project to distinct targets. L2/3 PNs send axons to both neighboring and distant cortical regions (Fame et al., 2011; Harris and Shepherd, 2015). Presumably they are important for integrating information across cortical areas and mediating higher order information processing. On the other hand, L5 PNs constitute a major source of cortical outputs to subcortical structures, projecting axons to regions such as the thalamus, the striatum, the midbrain, the pons and the spinal cord (O’Leary and Koester, 1993; Harris and Shepherd, 2015). Second, L5 and L2/3 PNs differ in cell body size and dendritic arborization. L2/3 PNs have smaller somata and more confined dendritic trees compared to L5 PNs (Larkman and Mason, 1990; Feldmeyer, 2012; Rojo et al., 2016). Apical dendrites of L5 PNs extend a greater distance than those of L2/3 PNs to reach the pial surface, sampling a greater area of the cortex (Spruston, 2008). Finally, L2/3 PNs have a significantly lower spontaneous and evoked action potential firing rate than L5 PNs (Petersen and Crochet, 2013). These structural and functional differences between L2/3 and L5 PNs are thought to support their diverse roles in information processing within neural circuits.

Neurons interconnect and communicate with each other at specialized sites called synapses. The postsynaptic sites of the majority of excitatory synapses reside on dendritic spines, tiny protrusions emanating from dendrites (Gray, 1959). Spines contain molecular components for synaptic signaling and plasticity, including ionotropic and metabotropic receptors, cytoskeletal and adaptor proteins, and various signaling molecules (Nimchinsky et al., 2002; Hotulainen and Hoogenraad, 2010; Sheng and Kim, 2011; Colgan and Yasuda, 2014; Levy et al., 2014). In the past two decades, transgenic mice expressing fluorescent proteins (Feng et al., 2000) and two-photon microscopy (Denk et al., 1990) have enabled tracking the dynamic formation and elimination of spines, which imply corresponding changes in synaptic connections, in living animals over time (Holtmaat and Svoboda, 2009; Fu and Zuo, 2011; Chen et al., 2014b). Longitudinal imaging of spine dynamics demonstrates that spine formation and plasticity is fundamental to the development and experience-dependent remodeling of neural circuits throughout the animal’s life (Trachtenberg et al., 2002; Zuo et al., 2005b; Holtmaat et al., 2006; Hofer et al., 2009; Xu et al., 2009; Yang et al., 2009; Tropea et al., 2010; Attardo et al., 2015). The majority of *in vivo* imaging studies on the structural dynamics of dendritic spines have so far focused on L5 PNs in the cerebral cortex. This is largely due to the ready availability of transgenic mouse lines that preferentially and strongly express fluorescent proteins (i.e., yellow (YFP) or green fluorescent protein (GFP)) in a putatively random subset of L5 PNs. In addition, most chronic live imaging work using these mouse lines have focused on the plasticity of spines in L1 of the cortex because of their optical accessibility. While these studies have revealed interesting spatiotemporal patterns of spine dynamics under various conditions, there is no guarantee that the conclusions are universally applicable rules. For example, inputs to upper-layer PNs are distinct from those to L5 PNs (Feldmeyer, 2012; Hooks et al., 2013); similarly, apical and basal dendrites of the same neuron may form synapses with distinct neuronal populations (Spruston, 2008; Feldmeyer, 2012; Oberlaender et al., 2012): all these may result in different rules for synaptic dynamics.

In this study, we compared the developmental and experience-dependent spine dynamics along apical dendritic tufts of L2/3 vs. L5 PNs. Specifically, we investigated whether and how their spine dynamics differ through postnatal development into adulthood, during adolescent forelimb-specific motor skill learning, and in response to neonatal sensory deprivation. Despite a handful of articles directly comparing L2/3 and L5 PN apical dendritic spine dynamics (Holtmaat et al., 2005; Hofer et al., 2009; Schubert et al., 2013; Ma et al., 2016; Yang et al., 2016), the behavior of spines under the conditions above has never been investigated systematically. Given the importance of motor skill learning and early sensory experience for brain development, such data will improve our knowledge on how brain circuits change in response to early experience. We found that the spine density and the intrinsic spine dynamics are significantly higher in L2/3 PNs than in L5 PNs in both adolescent and adult mice. Interestingly, L2/3 and L5 PNs respond differently to neonatal sensory deprivation and adolescent motor learning, suggesting a circuit-specific modulation of excitatory connections in the cortex by experience.

## Materials and Methods

### Experimental Animals

All animal care and experimental procedures were performed in accordance with protocols approved by the Institutional Animal Care and Use Committee (IACUC) at University of California, Santa Cruz. *Thy1*-YFP-H line mice (Feng et al., 2000) were purchased from Jackson Laboratory. Timed pregnant C57Bl/6 female mice were purchased from Charles River. Mice were group-housed in the UCSC animal facility, with 12 h light-dark cycle and access to food and water *ad libitum*. Both male and female mice were used in all experiments.

### *In Utero* Electroporation

*In Utero* electroporation (IUE) was performed as previously described (Saito and Nakatsuji, 2001) on E13.5 or E15.5 timed pregnant C57Bl/6 mice to label L5 or L2/3 PNs, respectively. The pCAG-GFP plasmid (Addgene #11150) was purified using the NucleoBond Extra Midi EF Kit (Clontech Laboratories). The plasmid was diluted to a final concentration of 1 μg/μl with sterile phosphate buffered saline (PBS) and colorized with 0.1% Fast Green (Sigma-Aldrich) dissolved at 37°C immediately prior to use. One to two microliters DNA plasmid was injected into the lateral ventricle (LV) through a pulled glass micropipette. Five pulses (25–30 V amplitude, 50 ms duration with 950 ms intervals) were delivered, targeting the motor or barrel cortex, using a platinum plate tweezers-type electrode connected to a square-pulse electroporator (CUY21, NEPA Gene).

### Immunofluorescence for Cortical Sections and Confocal Imaging

**Figure 1:**
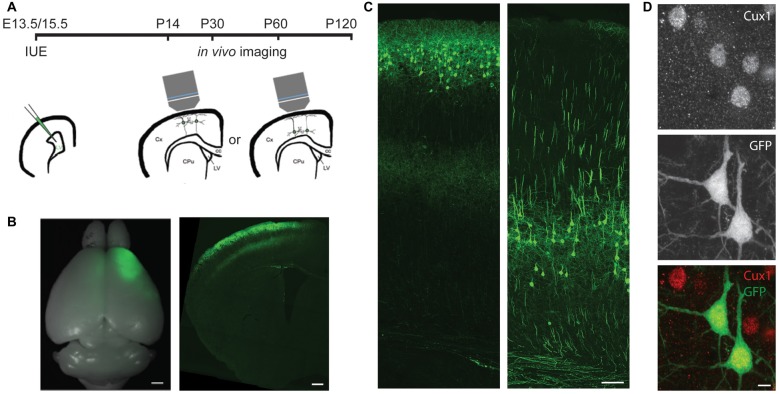
*In utero* electroporation (IUE) selectively labels cortical neurons in specific areas and layers. **(A)** Experimental design showing the timing of IUE and *in vivo* imaging. LV, lateral ventricle; Cx, cortex; CPu, striatum; cc, corpus callosum. **(B)** An example of E15.5 IUE targeting the motor cortex. Left: the whole brain. Right: a coronal section of one hemisphere. **(C)** Examples of L2/3 (left) or L5 (right) pyramidal neurons (PNs) in the motor cortex labeled by IUE. **(D)** An example of green fluorescent protein (GFP) neurons in the E13.5 electroporated brain co-labeling with Cux1 (Red). Scale bars: 1 mm (**B** left), 500 μm (**B** right), 100 μm **(C)** and 5 μm **(D)**.

The mouse was transcardially perfused with 4% paraformaldehyde (PFA) in 0.1 M PBS. Following perfusion, the brain was post-fixed in 4% PFA at 4°C overnight and cryoprotected with overnight incubation in 30% sucrose. The brain was then embedded in OCT medium and cryosectioned into 25 μm thick coronal sections. For immunostaining, sections were washed in PBS for 10 min, and incubated in blocking solution (5% goat serum, 0.01% Triton in PBS) for 15 min at room temperature in a humid chamber. Sections were then quickly washed in PBS and labeled with rabbit anti-Cux1 (1:1000; Santa Cruz Biotechnology) at 4°C overnight in a humid chamber. Sections were subsequently incubated with goat anti-rabbit secondary antibody conjugated with Alexa Fluor 594 (1:500; Life Technologies) in 0.1 M PBS for 2 h at room temperature. Finally, sections were washed in PBS and mounted with Fluoromount-G mounting medium (Southern Biotech). Confocal images were taken with a Leica SP5 confocal microscope with 10×/0.3 NA, 20×/0.75 NA and 63×/1.4 NA oil-immersion objectives. All images shown in Figure 1 are representative of at least three replications. Merging of different channels into multi-color images was performed with Adobe Photoshop.

For quantification of fluorescently labeled cells co-labeled with Cux1, wide-field images of brain sections were collected on a Zeiss Axio Imager M2 microscope with a 20×/0.8 NA objective using the Axiovision software and cells were manually counted in Stereo Investigator (MicroBrightField).

### *In Vivo* Transcranial Imaging and Data Analysis

Transcranial two-photon imaging and analysis of spine density and dynamics of apical dendritic tufts were performed as previously described (Zuo et al., 2005a). All images were analyzed using ImageJ. Spine density was calculated by dividing the number of spines by the length of the dendritic segment on which they reside. Only dendritic segments that lie within a single optical section are analyzed. Percentage of spines eliminated or formed was calculated as the number of spines eliminated or formed over the total spines counted in the images obtained during the first imaging session. The numbers of animals and spines analyzed under various experimental conditions are summarized in Supplementary Tables S1, S2. All data are presented as mean ± SEM. Mann-Whitney U test and Kruskal-Wallis rank sum test followed by *post hoc* multiple comparisons test were used for statistical analysis. *p* < 0.05 was considered significant.

**Figure 2:**
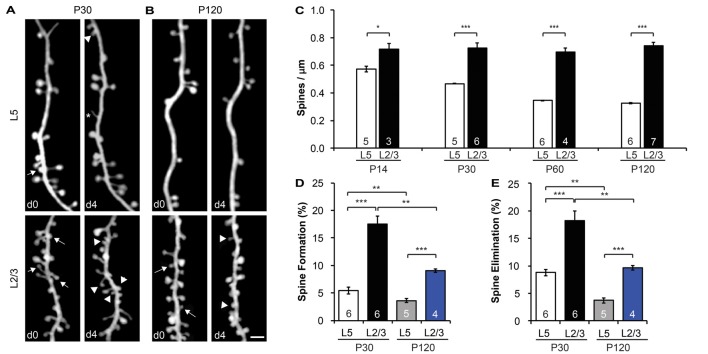
L2/3 PNs have higher spine density and dynamics than L5 PNs. **(A,B)** Repeated imaging of the same dendritic branches over 4-day intervals in the motor cortex of adolescent (P30) **(A)** and adult (P120) **(B)** mice. Arrows indicate eliminated spines, and arrowheads indicate newly formed spines. Filopodia are labeled by asterisks. Scale bar: 2 μm. **(C)** The spine density of L5 PNs undergoes a developmental decrease, whereas the spine density of L2/3 PNs remains constant from adolescence to adulthood. **(D,E)** Apical dendrites of L2/3 PNs display higher formation **(D)** and elimination **(E)** rates than L5 PNs in both adolescent and adult mice. Kruskal-Wallis rank sum test followed by *post hoc* multiple comparison was used for statistical analysis. *p < 0.05, **p < 0.01, ***p < 0.001. All data are presented as mean ± SEM. Numbers of mice analyzed are indicated in the figure.

Image processing for Figures 2A,B were performed as previously described (Xu et al., 2009). Briefly, we chose regions with sparsely labeled dendrites as examples and made maximum intensity projections of the image stack. The resulted images were then thresholded, Gaussian filtered and contrast-enhanced for presentation.

### Single-Pellet Reaching Task

Single-pellet reaching test was performed as previously described (Xu et al., 2009; Chen et al., 2014a). Briefly, the mouse was food-restricted to maintain 90% of the *ad libitum* weight during the experiment. A brief shaping phase was used to familiarize the mouse with the training chamber and task requirements, as well as to determine its limb preference. Thirty pellets were used for each training session. Reach attempts were scored and the success rate was calculated as the percentage of successful reaches over total reaches per session.

### Sensory Deprivation

Whisker trimming was performed as previously described (Lee et al., 2009). Mystacial vibrissae of both side whisker-pads were cut to skin level daily from postnatal day 0–7. Control mice were handled similarly without whisker trimming.

### Whisker-Dependent Texture Discrimination Task

Whisker-dependent texture discrimination test was performed as previously described (Wu et al., 2013; Chen and Zuo, 2015). Briefly, the mouse was habituated and tested in a testing arena (38 cm × 28 cm × 23 cm). On the testing day the mouse went through three phases: encoding (5 min), rest (5 min) and testing (3 min). In the encoding phase, the mouse was presented with two identically textured columns (3 cm × 3 cm × 9 cm). One of the columns was replaced with a new column of a different texture during testing phase. The amount of time spent actively investigating the columns was recorded and analyzed using EthoVision XT 10-Noldus software. Data are presented as mean ± SEM. Student’s *t*-test was performed to compare the percentage of time spent investigating the columns during testing. *p* < 0.05 was considered significant.

## Results

### Timed *In Utero* Electroporation Selectively Labels Cortical Neurons in Specific Areas and Layers

To specifically label L5 or L2/3 PNs, we electroporated plasmids encoding the GFP into the mouse cortex on either embryonic day (E) 13.5 or E15.5, respectively (Figures 1A–C). By adjusting the electrode position, we selectively targeted either the barrel or the motor cortex. None of the GFP+ cells in E13.5-electroporated brains co-labeled with Cux1, a marker for upper-layer neurons (Arlotta et al., 2005; Molyneaux et al., 2007), but the majority (>94%, 407 cells from three mice) of GFP+ cells in E15.5-electroporated brains did (Figure 1D). These data demonstrate our capability to target PNs of a particular cortical region in a layer-specific manner.

### L2/3 PNs have Higher Spine Density Along Apical Dendrites than L5 PNs and Lack Spine Pruning during Adolescent Development

As most excitatory synapses reside on spines (Gray, 1959), spine density is a good indicator of a neuron’s excitatory synaptic connectivity. To compare the spine density on apical dendrites of L2/3 vs. L5 PNs, we imaged dendritic segments in L1 of the motor cortex of both electroporated mice and YFP-H line mice with transcranial *in vivo* two-photon microscopy. We found that the spine density of L5 PNs was comparable between E13.5-electroporated mice (0.44 ± 0.04 spines/μm) and YFP-H mice (0.47 ± 0.01 spines/μm) at P30 (*p* > 0.6, Supplementary Figure S1A). However, the spine density along L2/3 PNs (0.73 ± 0.04 spines/μm) was almost twice that of L5 PNs at the same age (*p* < 0.001, Figures 2A,C). This difference in spine density between L2/3 and L5 PNs was also observed in the barrel cortex (*p* < 0.001, Supplementary Figure S1B). It is worth noting that the spine density of L5 and L2/3 PNs was comparable between barrel and motor cortices (*p* > 0.3, Supplementary Figure S1B). Furthermore, we found that spine density of L5 PNs decreased from postnatal day (P) 14 (early adolescent) until P120 (adulthood; Figure 2C), consistent with earlier findings in the sensory cortex (Grutzendler et al., 2002; Holtmaat et al., 2005; Zuo et al., 2005a). Given the developmental pruning of spines, the higher spine density observed on L2/3 PNs at P30 could be due to a slower or delayed spine pruning or a higher spine density to start with. To distinguish between these possibilities, we compared spine densities of L2/3 PNs and L5 PNs at three other ages (P14, P60 and P120; Figure 2C). We found no difference in spine density along L2/3 PNs among these age groups (*p* > 0.7), and spine densities of L2/3 PNs were significantly higher than that of L5 PNs at all ages examined (*p* < 0.05). In summary, apical dendrites of L2/3 PNs harbor intrinsically higher spine density than L5 PNs, but unlike L5 PNs, L2/3 PNs do not show spine pruning after P14.

### Apical Dendrites of L2/3 PNs Exhibit Higher Spine Dynamics than L5 PNs in Both Adolescent and Adult Mice

Time-lapse imaging has accumulated much evidence that synaptic connections are constantly formed and eliminated in the living brain, even in adulthood (Holtmaat and Svoboda, 2009; Chen et al., 2014b). To compare baseline spine dynamics on apical dendrites of L2/3 vs. L5 PNs, we followed the same dendritic segments over a 4-day interval and compared spine changes between imaging sessions at P30 and P120 (Figures 2A,B). We found that spines on L2/3 PNs are much more dynamic than spines on L5 PNs. In the motor cortex over a 4-day interval at P30, 17.5 ± 1.5% of spines were formed on L2/3 PNs, significantly higher than that of L5 PNs (5.5 ± 0.6%, *p* < 0.001; Figure 2D). Similarly, 18.2 ± 1.8% of spines on L2/3 PNs were eliminated over the same period, compared to 8.8 ± 0.6% on L5 PNs (*p* < 0.001, Figure 2E). In addition, our results revealed that L5 PNs have significantly higher spine elimination than formation (*p* < 0.05, Supplementary Figure S2A), consistent with the decrease in spine density during adolescent development (Holtmaat et al., 2005). In contrast, L2/3 PNs had balanced spine formation and elimination at P30 (*p* > 0.6), consistent with the lack of spine pruning in the adolescent brain (Supplementary Figure S2A).

We also found that spine dynamics of both L2/3 and L5 PNs slowed down in the adult brain. In the motor cortex at P120, spine formation and elimination rates of L2/3 PNs over 4 days were 9.0 ± 0.3% and 9.7 ± 0.4%, respectively, significantly lower than those measured at P30 (*p* < 0.01 for both, Figures 2D,E). Nevertheless, as in adolescence, these rates were still higher than corresponding ones of L5 PNs (3.6 ± 0.4% formation, 3.7 ± 0.4% elimination, *p* < 0.001 for both, Figures 2D,E). Importantly, L2/3 and L5 PNs had balanced spine formation and elimination at P120 (*p* > 0.5 for both, Supplementary Figure S2B), suggesting that spine density reaches a constant level for both PNs in adults.

### Motor Skill Learning-Induced Increase in Spine Dynamics Occurs in L5, but Not L2/3, PNs of the Motor Cortex

**Figure 3:**
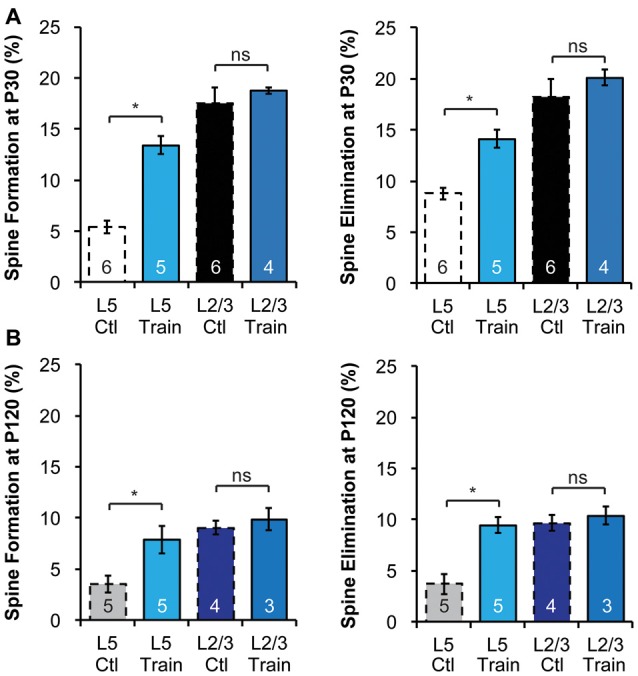
Motor learning does not enhance spine dynamics of L2/3 PNs in adolescence or adulthood. **(A,B)** Motor learning increases the spine dynamics of L5 PNs, but has no effect on spine dynamics of L2/3 PNs in both P30 adolescent **(A)** and P120 adult **(B)** mice. Kruskal-Wallis rank sum test followed by *post hoc* multiple comparison was used for statistical analysis. *p < 0.05. All data are presented as mean ± SEM. Numbers of mice analyzed are indicated in the figure.

The differences in baseline structural dynamics between L2/3 and L5 PNs prompted us to ask if experience-dependent spine plasticity could also differ. To do so, we trained mice to reach for single food pellets, a forelimb-specific motor-skill learning task (Xu et al., 2009), and imaged the contralateral motor cortex over a 4-day interval at P30 and P120 to determine spine dynamics changes. Consistent with earlier work (Xu et al., 2009), we found that motor-skill learning increased spine formation and elimination of L5 PNs at both P30 and P120 (Figures 3A,B). At P30, 13.4 ± 0.9% and 14.1 ± 0.9% spines were formed and eliminated, respectively, on the apical dendrites of L5 PNs in mice undergoing daily training, significantly higher than those in control mice (*p* < 0.05 for both, Figure 3A). In contrast, 18.7 ± 0.3% and 20.1 ± 0.8% spines were formed and eliminated, respectively, on the apical dendrites of L2/3 PNs during motor-skill learning, not significantly different from those in control mice (*p* > 0.2 for both, Figure 3A). We observed a similar effect in adulthood as well. While L5 PNs responded to learning with elevated formation (7.9 ± 0.6%) and elimination (9.5 ± 0.3%; *p* < 0.05 for both compared to controls), L2/3 PNs failed to do so (9.9 ± 0.6% formation and 10.4 ± 0.5% elimination with training, *p* > 0.2 for both compared to controls, Figure 3B).

### Early Postnatal Sensory Deprivation Impairs Whisker-Dependent Textural Discrimination and Alters Cortical Spine Dynamics in a Layer-Specific Manner in Adolescent Mice

**Figure 4:**
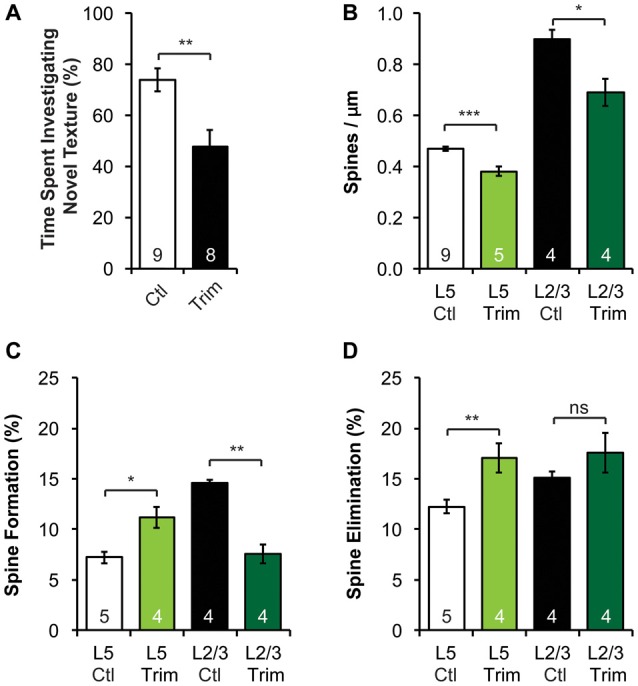
Neonatal sensory deprivation alters whisker-dependent behavior, spine density and dynamics of L5 and L2/3 PNs in the barrel cortex. **(A)** Sensory-deprived mice have defective whisker discrimination at P30. **(B)** Whisker-trimmed mice have significantly lower spine density on both L5 and L2/3 PNs, compared to age-matched controls. **(C,D)** Spine formation and elimination are altered in both L5 and L2/3 PNs in the trimmed mice. Student’s *t*-test **(A)** and Kruskal-Wallis rank sum test followed by *post hoc* multiple comparison **(B–D)** were used for statistical analysis. *p < 0.05, **p < 0.01, ***p < 0.001. All data are presented as mean ± SEM. Numbers of mice analyzed are indicated in the figure.

Sensory experience during early postnatal life is crucial for the proper development of neuronal morphology and sensory acuity in rodents (Hubel and Wiesel, 1964; Carvell and Simons, 1996; Shoykhet et al., 2005; Lee et al., 2009; Wimmer et al., 2010; Chen C.-C. et al., 2012; Chen et al., 2015; Erzurumlu and Gaspar, 2012; Papaioannou et al., 2013). To determine if neonatal sensory deprivation alters sensory processing later in life, we bilaterally trimmed the whiskers of pups during the first postnatal week (i.e., from P0 to P7), before active whisking starts (Landers and Philip Zeigler, 2006; Erzurumlu and Gaspar, 2012). We then waited for the whiskers to grow back to full length (*p* > 0.3, Supplementary Figure S3A) and assessed whisker function using the whisker-dependent textural discrimination task (Wu et al., 2013) at P30. We found that control mice spent significantly more time approaching the column with the novel texture to the column with the habituated texture (*p* < 0.01, Supplementary Figure S3C). In contrast, trimmed mice spent equal amount of time investigating novel and habituated texture (*p* > 0.4, Supplementary Figure S3D). Together, trimmed mice spent a smaller fraction of time approaching the column with a novel texture compared to control (*p* < 0.01, Figure 4A). It is important to note that there was no significant difference in the amount of time spent investigating the columns during encoding between control and trimmed mice (*p* > 0.6, Supplementary Figure S3B), suggesting no defect in exploration activity. These data suggest that early sensory experience is crucial for the development of normal whisker-dependent textural discrimination ability.

As the integrity of the barrel cortex is necessary for this texture discrimination task (Chen and Zuo, 2015), we next asked if neonatal whisker trimming alters synaptic connectivity and dynamics in the barrel cortex. To do so, we imaged apical dendrites of L2/3 and L5 PNs in the barrel cortex over a 7-day interval of both control and trimmed mice at P30. We found that the spine density of both L2/3 and L5 PNs in trimmed mice were significantly lower than that of controls (Figure 4B, 0.47 ± 0.01 vs. 0.38 ± 0.02 spines/μm for L5 PNs, *p* < 0.001; 0.90 ± 0.04 vs. 0.69 ± 0.05 spines/μm for L2/3 PNs, *p* < 0.05). Following the same dendrites over time, we also found that L5 PNs in trimmed mice had significantly higher spine formation and elimination than in control mice (formation: 11.2 ± 1.0% vs. 7.2 ± 0.6%, *p* < 0.05; elimination: 17.0 ± 1.5% vs. 12.3 ± 0.7%, *p* < 0.01; Figures 4C,D). Interestingly, L2/3 PNs responded to trimming differently from L5 PNs, with trimmed mice showing lower spine formation than control mice (7.6 ± 0.9% vs. 14.6 ± 0.3%, *p* < 0.01), but comparable spine elimination (17.6 ± 2.0% vs. 15.1 ± 0.6%, *p* > 0.7; Figures 4C,D). Together, these results suggest that neonatal sensory deprivation differentially affects the spine dynamics of PNs whose cell bodies reside in different layers, suggesting layer-specific rearrangements of excitatory connectivity.

## Discussion

Combining IUE and *in vivo* two-photon imaging, we examined the structural plasticity of apical dendritic tufts of either L2/3 or L5 PNs in the motor and barrel cortices. Our results show that spine density and baseline spine dynamics are significantly higher in L2/3 PNs than in L5 PNs at all ages and regions examined. The higher spine density in L2/3 PN has also been previously reported in adults (Holtmaat et al., 2005). Interestingly, spine density obtained *in vivo* varies among studies. Our measured spine density is consistent with some earlier studies (Zuo et al., 2005b; Yu et al., 2013; Hayashi-Takagi et al., 2014), but slightly higher than the data reported in other publications (Trachtenberg et al., 2002; Holtmaat et al., 2005). We found that while L5 PNs undergo a developmental decrease in the number of spines, due to significantly higher spine elimination compared to formation as shown previously (Holtmaat et al., 2005; Zuo et al., 2005a,b). Interestingly, L2/3 PNs maintain a constant number of spines as the animal develops. As pruning of supernumerary synapses is believed to be prevalent in the maturation of the nervous system (Lichtman, 1995; Lichtman and Colman, 2000; Pentajek et al., 2011), it is important for future research to determine whether our study missed an earlier phase (before P14) of spine pruning, or spine pruning indeed does not occur in L2/3 PNs. The difference in baseline spine dynamics may be due to different circuit connections of L2/3 and L5 PNs (Anderson et al., 2010; Feldmeyer, 2012; Hooks et al., 2013; Kaneko, 2013). It may also be a consequence of different neuronal activities of L2/3 and L5 PNs (Petersen and Crochet, 2013). As most brain energy is spent on synaptic transmission, the difference in spine density and dynamics of L2/3 and L5 PNs may arise from the differences in their metabolic capacity (Harris et al., 2012).

We observed that L2/3 PNs fail to increase spine dynamics during motor learning. This result is consistent with a previous study showing that monocular deprivation increases spine formation and leads to higher spine density on the apical tufts of L5, but not L2/3, PNs in the binocular region of the mouse visual cortex (Hofer et al., 2009). A more recent work revealed pathway-specific increases in the formation of lateral amygdala axon boutons and dendritic spines of L5 PNs in the auditory cortex during fear conditioning, but no change in spine dynamics of L2/3 PNs (Yang et al., 2016). Given their high baseline spine dynamics, L2/3 PNs may have already reached the metabolic ceiling under baseline conditions, so cannot support higher spine dynamics. However, lack of spine dynamics change does not exclude L2/3 PNs from participating in motor learning. In fact, studies have shown L2/3 PNs are responsive during motor skill learning. For example, *in vivo* calcium imaging has revealed a convergence of L2/3 PN activity as the animal perfects its motor behavior (Peters et al., 2014). Furthermore, motor skill learning occludes LTP between L2/3-L2/3 connections and enhances LTD thereof in the motor cortex of rats (Rioult-Pedotti et al., 2000). These results suggest that motor learning may affect L2/3 PN connections via synaptic strengthening and weakening, rather than spine generation and removal. On the other hand, a recent study reports that spine dynamics on L2/3 PNs increases following a single session of treadmill training (Ma et al., 2016). This could be due to the different behavioral paradigms employed in this study and our work, which may involve different cortical circuits and thus evoke different spine remodeling patterns.

Many studies have shown that sensory experiences profoundly impact the organization and development of sensory cortices (Carvell and Simons, 1996; Majewska and Sur, 2003; Sadaka et al., 2003; Fox and Wong, 2005; Holtmaat et al., 2006; Lee et al., 2009; Briner et al., 2010; Popescu and Ebner, 2010; Tropea et al., 2010). Our results support this idea by showing that neonatal sensory deprivation leads to altered spine density/dynamics and defective whisker-dependent behavior. Our study, together with previous *in vivo* imaging studies, depicts a complex picture of sensory deprivation in the sensory cortex: the impact depends on the type of manipulation, the time window of manipulation, and the type of neurons (Fu and Zuo, 2011; Medini, 2014). In the visual cortex, dark rearing increases spine motility on L5 PNs (Tropea et al., 2010), and monocular deprivation increases spine formation on L5 PNs in the binocular zone (Hofer et al., 2009). Recent work also reveals that, while the dynamics of spines on L2/3 PNs in the visual cortex does not change in response to monocular deprivation, the proportion of clustered dynamic spines increases (Chen J. L. et al., 2012), and inhibitory synapses on spines are repeatedly assembled and removed (Villa et al., 2016). In the somatosensory cortex, trimming all whiskers decreases spine pruning (Zuo et al., 2005b), whereas chessboard trimming stabilizes new spines and destabilizes persistent spines in L5 PNs with complex apical tufts (Holtmaat et al., 2006). On the other hand, sensory deprivation via follicle removal has been shown not to significantly alter L5 or L2/3 spine density and turnover, but to increase new persistent spine formation of L2/3 PNs (Schubert et al., 2013). While the above studies focused on the effect of sensory deprivation on adolescent and adult spine plasticity, our work focused on the delayed effects of neonatal sensory deprivation. Specifically, neonatal (P0–7) bilateral whisker trimming decreases spine density of both L5 and L2/3 PNs. It is possible that the decrease in spine density is due to a reduction in axonal branches from the thalamus (Wimmer et al., 2010), which may result in an overall decrease in excitatory inputs to the apical tufts. In addition to reduction in spine density in apical tufts of L5 and L2/3 PNs, we observed layer-specific changes in spine dynamics. Under our experimental paradigm, it is understandable that in response to neonatal whisker trimming L5 PNs exhibit higher spine formation and elimination (Figure 4), mimicking an immature stage of the developing brain. However, it is puzzling that L2/3 PNs in the trimmed mice decrease spine formation without changes in spine elimination. The difference in spine dynamics of L5 and L2/3 PNs in response to neonatal whisker trimming suggests that there are functional differences in sensory processing between L5 and L2/3 PNs. As a recent study challenges the canonical model of information flow in the rodent barrel cortex and questions the functional role of L2/3 PNs in sensory processing (Constantinople and Bruno, 2013), more studies are necessary to understand the synaptic organization and plasticity of L2/3 PNs.

In summary, our data suggest different dynamic rules governing experience-dependent structural plasticity of apical dendritic spines of PNs in different cortical layers. However, we cannot prove that new spines observed in this study all have synapses. Indeed, previous studies combining *in vivo* optical imaging with correlative electron microscopy or fluorescent labeling of synaptic proteins such as PSD95 have shown that not all new spines have synapses (Knott et al., 2006; Cane et al., 2014). In addition, previous studies have revealed that many of the new spines are transient (Xu et al., 2009; Yang et al., 2009), calling into question their long-term functional significance. Furthermore, the presynaptic partners of these spines remain elusive. Thus, a comprehensive understanding of the reorganization of synaptic circuits requires concurrent imaging of pre- and post-synaptic elements as illustrated by a recent study on the amygdalocortical circuit (Yang et al., 2016), or correlative light and electron microscopy (Knott et al., 2006).

## Author Contributions

MT and XY performed *in vivo* imaging and spine analyses. MT and LSJ performed immunohistochemistry and behavioral experiments, and analyzed the data. XY made the examples of spine images. MT made all figures. MT, JL and YZ designed the experiment and wrote the manuscript.

## Conflict of Interest Statement

The authors declare that the research was conducted in the absence of any commercial or financial relationships that could be construed as a potential conflict of interest.
