# Supplementary material for: Pyramidal Neurons in Different Cortical Layers Exhibit Distinct Dynamics and Plasticity of Apical Dendritic Spines
Source: Front Neural Circuits. 2017 Jun 19;11:43. doi: 10.3389/fncir.2017.00043 (PMC5474458; doi:10.3389/fncir.2017.00043)
Supplement: Supplementary file 1 [file Presentation_1.pdf]

Supplementary Material

Pyramidal neurons in different cortical layers exhibit distinct dynamics and plasticity of apical dendritic spines

Michelle Tjia, Xinzhu Yu, Lavpreet S. Jammu, Ju Lu, and Yi Zuo*

*** Correspondence:** Yi Zuo: yizuo@ucsc.edu

1 Supplementary Figures and Tables

1.1 Supplementary Tables

Supplementary Table 1. The percentage of spines eliminated and formed over various intervals under different experimental conditions. Training: single-pellet reaching task, Trimming: neonatal sensory deprivation (P0-7). Data are presented as mean ± s.e.m., MC: motor cortex, BC: barrel cortex

Imaging Intervals	Layer	Conditions	Spine Formation (%)	Spine Elimination (%)	Spine Number	Animal Number
**P30**						
4 days	L5, MC	Control	5.5 ± 0.6	8.8 ± 0.6	749	6
		Training	13.4 ± 0.9	14.1 ± 0.9	719	5
4 days	L2/3, MC	Control	17.5 ± 1.5	18.2 ± 1.8	782	6
		Training	18.7 ± 0.3	20.1 ± 0.8	481	5
7 days	L5, BC	Control	7.2 ± 0.6	12.3 ± 0.7	759	5
		Trimming	11.2 ± 1.0	17.0 ± 1.5	526	4
7 days	L2/3, BC	Control	14.6 ± 0.3	15.1 ± 0.6	484	4
		Trimming	7.6 ± 0.9	17.6 ± 2.0	509	4
**P120**						
4 days	L5, MC	Control	3.6 ± 0.4	3.7 ± 0.4	619	5
		Training	7.9 ± 0.6	9.5 ± 0.3	806	5
4 days	L2/3, MC	Control	9.0 ± 0.3	9.7 ± 0.4	587	4
		Training	9.9 ± 0.6	10.4 ± 0.5	469	3

Supplementary Table 2. Spine density under different experimental conditions. Data are presented as mean ± s.e.m. MC: motor cortex, BC: barrel cortex

Age	Cortex	Layer	Conditions	Spine Density (/µm)	Spine Number	Animal Number
P14	MC	L5	Control	0.57 ± 0.02	773	5
		L2/3	Control	0.72 ± 0.04	637	3
P30	MC	L5	Control	0.47 ± 0.00	926	5
		L2/3	Control	0.73 ± 0.04	1249	6
P60	MC	L5	Control	0.35 ± 0.00	1284	6
		L2/3	Control	0.70 ± 0.03	767	4
P120	MC	L5	Control	0.33 ± 0.01	665	5
		L2/3	Control	0.74 ± 0.02	1101	7
P30	BC	L5	Control	0.47 ± 0.01	1117	9
		L5	Trim	0.38 ± 0.02	607	5
P30	BC	L2/3	Control	0.90 ± 0.04	486	4
		L2/3	Trim	0.69 ± 0.05	484	4
P30	BC	L5	IUE	0.44 ± 0.04	850	7

1.2 Supplementary Figures

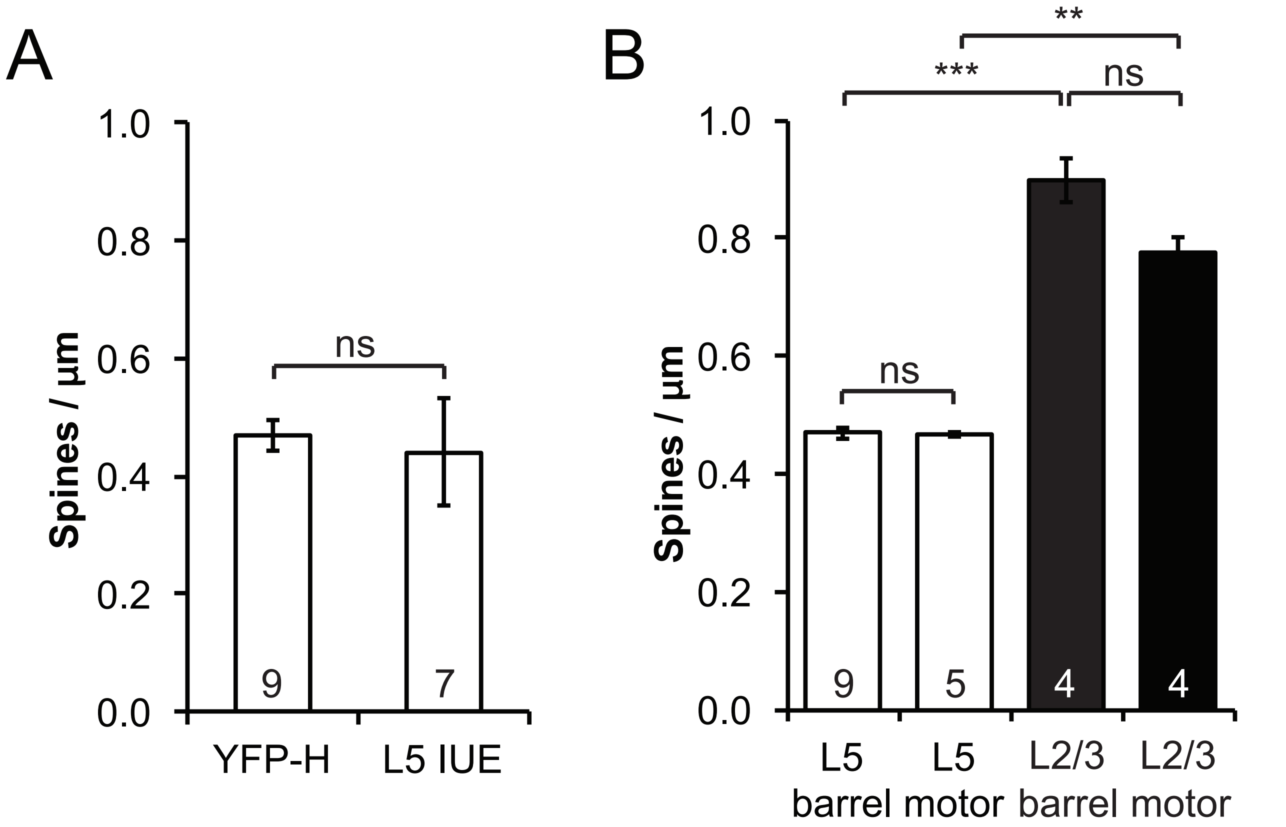

Supplementary Figure 1. Comparison of spine density in different cortical layers and regions. (A) Spine densities of apical dendrites of L5 PNs are comparable between E13.5 electroporated mice and YFP-H line mice. Mann-Whitney U test. **(B)** The spine density of PNs in the same layer is comparable between motor and barrel cortices, with higher density in L5 PNs compared to L2/3 PNs. Kruskal-Wallis rank sum test with post-hoc multiple comparisons was used for statistical analysis. $^{**}p < 0.01$, $^{***}p < 0.001$. Data are presented as mean ± s.e.m. Numbers of mice analyzed are indicated in the figure.

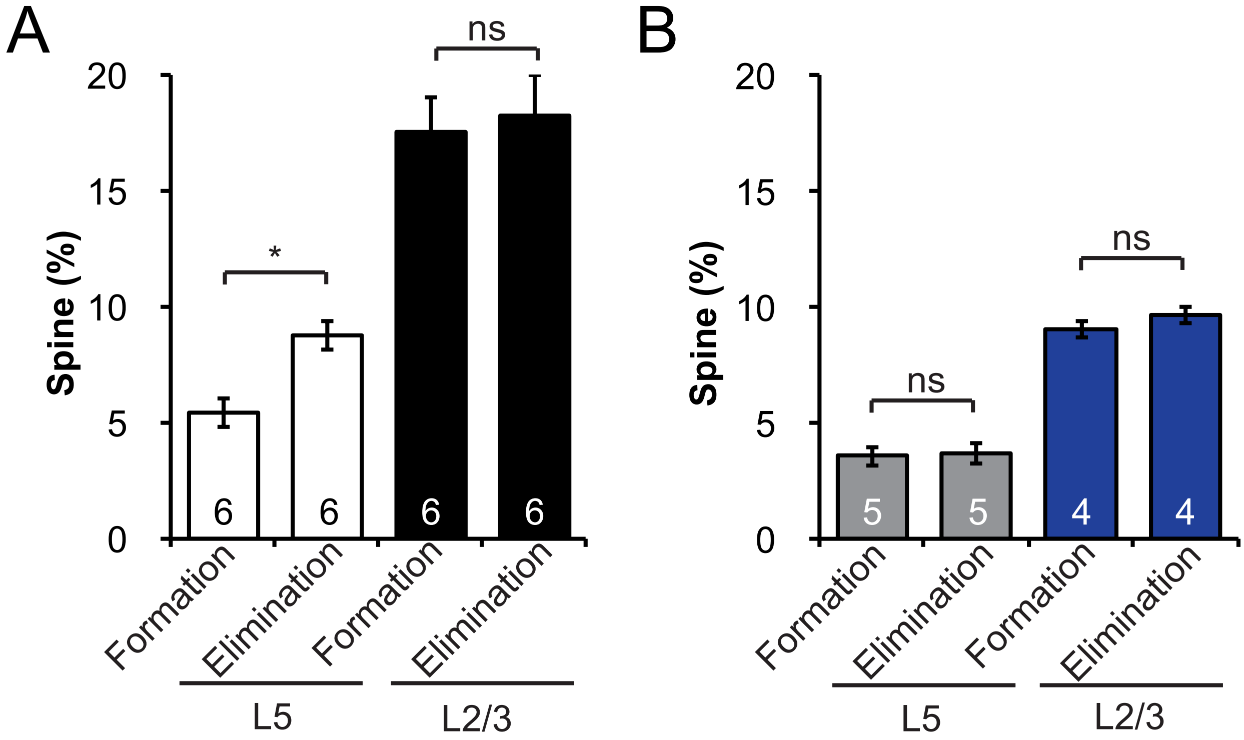

Supplementary Figure 2. Comparison of spine formation and elimination at different ages. (A) Significantly more spines were eliminated than formed along apical dendrites of L5 PNs, but not L2/3 PNs, over 4 days in P30 motor cortex. **(B)** Comparable amount of spines were formed and eliminated along the apical dendrites of both L2/3 and L5 PNs over 4 days in the P120 motor cortex. Kruskal-Wallis rank sum test with post-hoc multiple comparisons was used for statistical analysis. *$p < 0.05$. Data are presented as mean ± s.e.m. Numbers of mice analyzed are indicated in the figure.

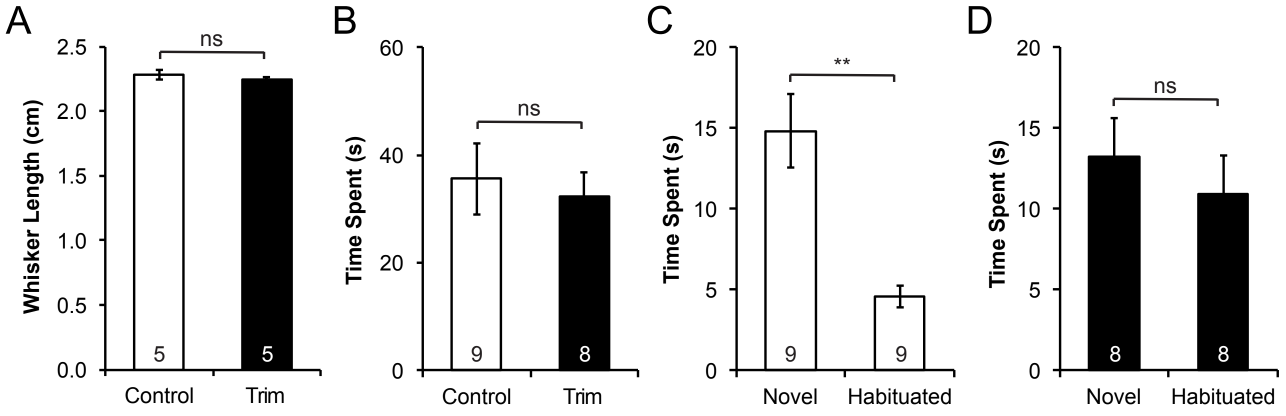

Supplementary Figure 3. Mice with neonatal whisker trimming fail to distinguish novel and habituated textures, despite normal whisker length and encoding behavior. (A) Whiskers of P0-7 trimmed mice grew back to control length at P30. **(B)** Control and trimmed mice spent comparable investigating time during encoding phase. **(C, D)** During testing phase, control mice spent significantly more time investigating the novel textured object compared to the habituated object **(C)**, whereas trimmed mice spent comparable time investigating novel and habituated textures **(D)**. Student *t*-test was used for statistical analysis. **$p < 0.01$. Data are presented as mean ± s.e.m. Numbers of mice analyzed are indicated in the figure.
